# An Accelerator Design Using a MTCA Decomposition Algorithm for CNNs

**DOI:** 10.3390/s20195558

**Published:** 2020-09-28

**Authors:** Yunping Zhao, Jianzhuang Lu, Xiaowen Chen

**Affiliations:** College of Computer, National University of Defense Technology, Changsha 410073, China; zhaoyunping@nudt.edu.cn (Y.Z.); xwchen@nudt.edu.cn (X.C.)

**Keywords:** CNNs accelerator, parallel computing algorithm, hardware architecture

## Abstract

Due to the high throughput and high computing capability of convolutional neural networks (CNNs), researchers are paying increasing attention to the design of CNNs hardware accelerator architecture. Accordingly, in this paper, we propose a block parallel computing algorithm based on the matrix transformation computing algorithm (MTCA) to realize the convolution expansion and resolve the block problem of the intermediate matrix. It enables high parallel implementation on hardware. Moreover, we also provide a specific calculation method for the optimal partition of matrix multiplication to optimize performance. In our evaluation, our proposed method saves more than 60% of hardware storage space compared with the *im2col*(image to column) approach. More specifically, in the case of large-scale convolutions, it saves nearly 82% of storage space. Under the accelerator architecture framework designed in this paper, we realize the performance of 26.7GFLOPS-33.4GFLOPS (depending on convolution type) on FPGA(Field Programmable Gate Array) by reducing bandwidth and improving data reusability. It is 1.2×–4.0× faster than memory-efficient convolution (MEC) and *im2col*, respectively, and represents an effective solution for a large-scale convolution accelerator.

## 1. Introduction

At present, CNNs are widely used in image classification [1], target recognition [2,3], and semantic segmentation [4,5]. CNNs are essentially composed of a convolution layer, RELU layer (Rectified Linear Unit), pooling layer, and full connection layer. In the CNNs model, the convolution layer’s amount of calculations accounts for more than 85% of the total calculation [6], which brings huge workload. In addition, with the deepening of CNNs, the time complexity and space complexity of CNNs realized by software are increasing. The scheme of CNNs based on software cannot meet the current high-speed application requirements. In order to solve this problem, accelerator design schemes based on various platforms are proposed, such as graphics processing unit (GPU) [7], or customized application specific integrated circuits (ASIC) [8,9,10,11], field programmable gate arrays (FPGA), and other hardware to complete the acceleration of CNNs. However, due to the problems of power consumption, development cost, and cycles, the research and development of GPU and ASIC are largely limited.

Recently, most of the existing CNNs accelerators on FPGA have been proposed [12,13,14]. These designs only focus on the improvement of performance, and ignore the overhead of energy and storage. However, the power consumption restricts their use in an embedded platform or mobile devices directly, and the overhead of storage leads to increase the requirement of hardware, thereby increasing the cost. These two aspects bring great challenges to the promotion of accelerators.

In this paper, we propose a CNNs accelerator design using a MTCA [15] decomposition algorithm. The convolution is divided into blocks and expanded by the algorithm, so as to realize the highly parallel computing of each matrix multiplication array (MMA) in the accelerator. We implement it on an FPGA platform with the systolic array architecture [16]. Generally speaking, the main advantages of this architecture are as follows.

Firstly, it optimizes the convolution expansion and storage mode by utilizing a block parallel computing algorithm based on MTCA. Not only does the operational efficiency improve, it also reduces the storage cost of the intermediate matrix expanded by convolution. Moreover, the MTCA-based block computing algorithm can ensure the high parallel operation of the calculation module and achieve optimal algorithmic efficiency.

Secondly, a matrix multiplication array (MMA) based on a systolic parallel architecture is adopted to ensure a high parallelism and independence in both the MMA and the internal PE (Processing Element), which gives the accelerator design good scalability. This approach can avoid the transmission of partial-sum between PEs, optimize the internal pipeline, and achieve efficient processing. With the support of the specific dataflow mode, the kernel level matrix operation is realized via row calculation, whose data are fetched from row vectors of matrix. It can avoid the access to the column vector data and the transposition of the matrix. As a result, our design reduces the control difficulty and hardware costs.

The organizational structure of the paper is as follows. Section II summarizes the related work. Section III introduces and explains the MTCA algorithm. Section IV introduces the architecture of accelerator in detail. Section V provides the implementation results and comparison. Section VI is the conclusion.

## 2. Motivation and Related Work

In this section, we introduce *im2col*, MEC, MTCA algorithms. We also introduce the related work and challenges.

### 2.1. Im2col, MEC, MTCA Algorithm

**Figure 1:**
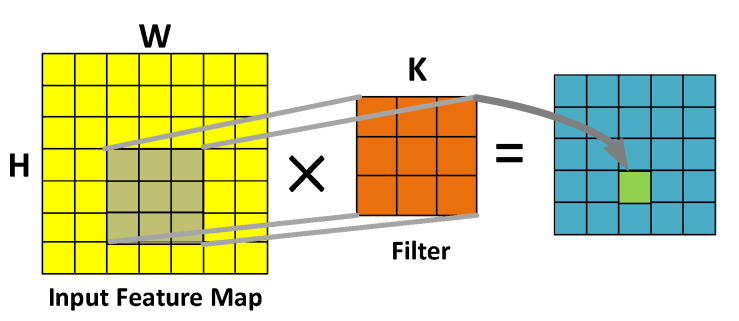
Calculation process of conventional convolutions.

Figure 1 shows the operational processes of conventional convolution. The operation process of conventional convolution involves executing the inner product between the gray part of the input feature map and the filter (see Figure 1); the result is an element of the output feature map, while the sliding window can obtain the final output feature map.

**Figure 2:**
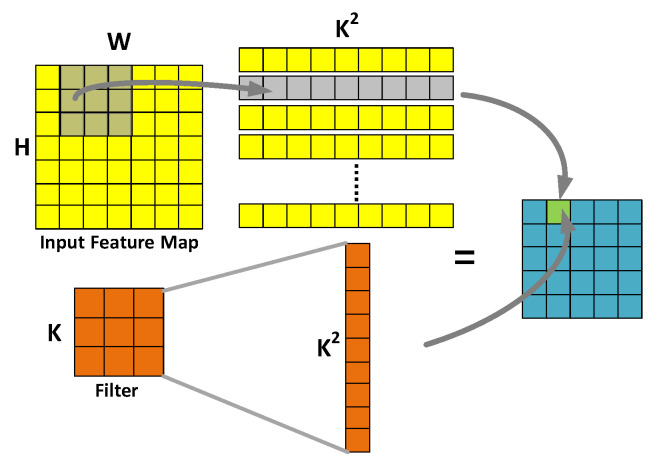
Calculation process of *im2col*.

For the *im2col* process described in Figure 2, both the input feature map and filter are spread by rows. The final output feature map is obtained by two intermediate process matrix operations. Although the intermediate matrix increases the memory cost, it can accelerate the convolution by using the matrix vector multiplication library, and finally improve the computational performance.

**Figure 3:**
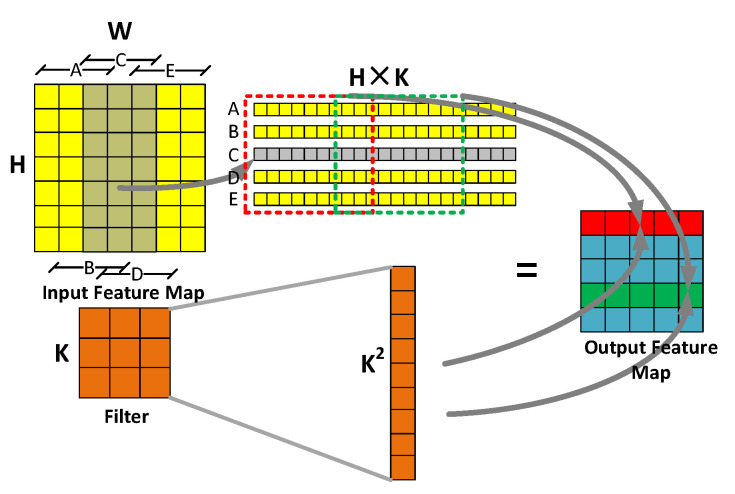
Calculation process of MEC.

For MEC, moreover (see Figure 3), the input feature map is divided into sections A–E longitudinally according to the size of H × K, after which each segment is expanded to obtain the intermediate matrix row by row (The gray part of Figure 3). The filter is expanded by row, which is the same as *im2col*. In this case, we obtain two intermediate matrices with size (5,H × K) and (K^2,1). Taking out a part of the intermediate matrix in a (5,K^2) window, and perform matrix multiplication with the intermediate matrix of filter. The result is a row of output feature map elements (The red or green parts of Figure 3), while the sliding window with step size of K can obtain the final output feature map.

**Figure 4:**
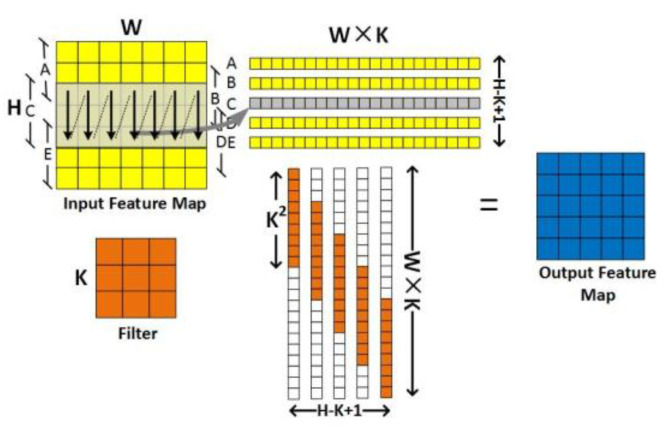
The operation process of MTCA.

The operation process of the MTCA algorithm is illustrated in Figure 4. Different from MEC, the input feature map is horizontally divided into sections A–E according to the size of K × W, then expanded to obtain a matrix of (H × K,H-K + 1). The data expansion method of each part of A–E is shown by the arrow in the figure. Based on the *im2col* expansion, the filter is then expanded into a matrix of (H-K + 1,H × K) size by zero; subsequently, the final output feature map is obtained by multiplying the two intermediate matrices.

### 2.2. Related Work

Previous studies have proposed a number of designs about CNNs accelerator architecture. The DianNao [17,18] series achieved high performance by improving the parallel computing method, and optimizing the dataflow model. Eyeriss [19] reduced data mobility by maximizing local data reuse, and implementing data gating. However, the development cycle of them are too long to adapt the rapid development of CNNs, and they did not study the large-scale convolution computation.

You Huang [20] realized the high-performance for large-scale convolution by optimization of the matrix-multiplication computing architecture. Junyang Zhang [21] proposed a large-scale convolution parallel computing method that realized high-performance CNN acceleration on FT2000 architecture. However, none of them optimize the storage efficiency of convolution expansion. Jing. Shen [22] proposed a design to balance storage bandwidth and computational speed, the convolutional layer is subdivided into granular tasks and executed to mask the time of accessing external storage, which outperforms traditional accelerators. Chaoyang Zhu [23] propose a sparse wise dataflow to skip the cycles of processing multiply-and-accumulates (MACs) with zero weights and exploit data statistics to minimize energy through zeros gating to avoid unnecessary computations, which provides high speedup and energy efficiency. Maurizio Capra [24] introduces what a hardware accelerator is, and what are its main components, followed by the latest techniques in the field of dataflow, reconfigurability, variable bit-width, and sparsity.

In addition, existing studies have proposed a number of designs about improving storage efficiency in terms of algorithms. M. Cho [25] proposed a memory-efficient convolution (MEC) algorithm to reduce the overhead of storage. Yulin Fang [15] proposed a convolution optimization algorithm based on computing unified device architecture (CUDA) acceleration library. However, the logic of piecewise convolution control is complex, and the address of the segmented matrix is not aligned. It is not conducive to hardware implementation.

### 2.3. Motivation

In summary, in most existing designs, it is rarely involved in the following three aspects. How to reduce the storage overhead of the convolution expansion? How to reduce the difficulty of memory read-write control? How to accelerate large-scale convolution? We proposed the block algorithm based on MTCA to achieve large-scale convolution computation. Compared with the *im2col*, MEC, MTCA, we improve the operation performance, save hardware resources, and improve the computational efficiency by realizing the optimal blocking. In addition, we reduce the difficulty of reading and writing control, which is especially important in large-scale convolution.

## 3. Algorithm Analysis and Mapping

### 3.1. A Design of Block Parallel Computing Algorithm Based on MTCA

Although the MEC algorithm also introduces the intermediate matrix, it saves 51.2% memory space than *im2col*. However, the MEC also has some shortcomings, that is, the logic of the matrix partition is complex and the address space of the small matrix is not aligned, which makes it more difficult to implement the algorithm both in hardware and software.

Compared with MEC, MTCA has a larger intermediate transformation matrix, simpler control logic, and is easier to implement in parallel computing on hardware to achieve better performance. However, there will be a large number of elements with a value of zero in the intermediate process matrix expanded by the filter, while the proportion of non-zero elements is small. During the actual computing process, this will not only increase the operational load, but will also consume a large amount of hardware storage resources. At the same time, given the increasing size of the input feature map and filter, this setup is inconvenient for parallel computation in hardware implementation. Accordingly, in order to solve the above problems, we propose a block parallel computing algorithm based on MTCA.

**Figure 5:**
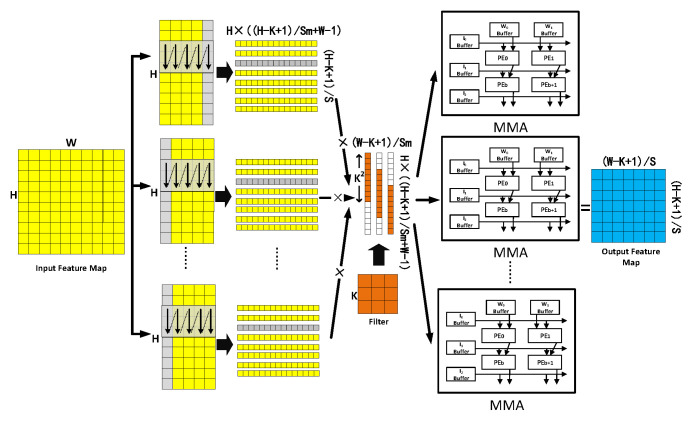
The block parallel computing algorithm based on MTCA.

As shown in Figure 5, the input feature map is longitudinally divided into m blocks, and each block is calculated via MTCA. During this process, whether or not each block should be supplemented is determined with reference to the number of blocks m. If the block size, W⁄m of the input feature map is less than (m−K+1)⁄(S(m+K−1)), each block matrix needs to be filled according to Equation (1) below; here, the capacity size is n, while S denotes the window sliding step size. MTCA:

(1) n=((W−K+1)/S×m+W−1)−W/m

Apart from the filling content of the first block, which comes from the back part of the input feature map, the filling contents of the other blocks come from the front part of the input feature map (marked in gray in Figure 5). According to the sliding size and the number of blocks, the filter fills to (H×(H−K+1)/(S×m+W−1), (w−k+1)/S×m) with 0 according to the MTCA. The intermediate process matrix of each block is assigned to each MMA in order to perform matrix-multiplication with the intermediate process matrix of the filter. The final output feature map is obtained via the final combination.

### 3.2. Computation Method of PE Based on Vector Single Instruction Multiple Data (SIMD) Technology

The computing performance of the accelerator is primarily determined by the computing power of each MMA. To reduce the idle time and pauses caused by memory access between the MMAs and the internal PEs, this paper uses a calculation method based on GEMM (General Matrix Multiplication) to realize the block calculation process by row. For the intermediate process matrix multiplication O = IW, the calculation method outlined in Equation (2) below is usually used:

(2) $$O_{\mathrm{ab}}=\overset{k-1}{\underset{l=0}{\sum }}I_{\mathrm{al}}\times W_{\mathrm{lb}},\;a=0,\;1,\;\ldots ,\;m-1;b=0,\;1,\ldots ,\;n-1$$

The intermediate process matrix W and output matrix O of the filter is divided into blocks according to row; the row vectors are $w_{b}$ and $o_{a}$, respectively. Equation (2) can then be transformed into Equation (3), as follows:

(3) $$\{\begin{array}{c} o_{0}=i_{00}w_{0}+i_{01}w_{1}+\ldots +i_{0k-1}w_{k-1}\\ o_{1}=i_{10}b_{0}+i_{11}w_{1}+\ldots +i_{1k-1}w_{k-1}\\ \ldots \\ o_{m-1}=i_{m-10}w_{0}+i_{m-10}w_{1}+\ldots +i_{m-1k-1}w_{k-1} \end{array}$$

**Figure 6:**
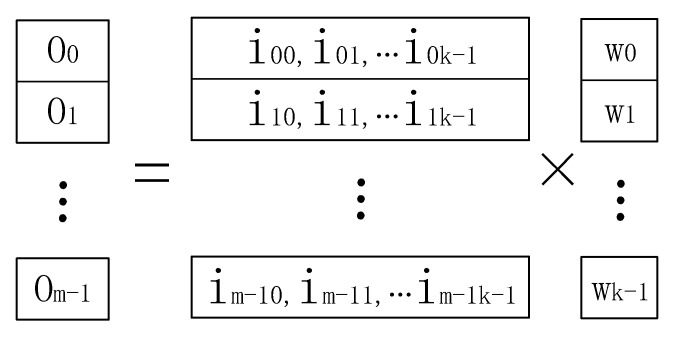
The method of calculating matrix multiplication by row.

**Figure 7:**
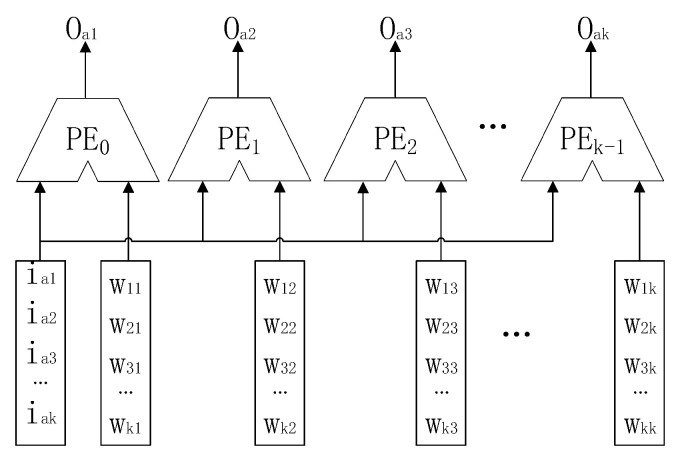
Realization of matrix multiplication based on vector SIMD technology.

As shown in Figure 6, in a departure from the conventional method, the vector calculation process [26] expands the b-th element of the line taken from the input intermediate process matrix I into a vector with the same value. As illustrated in Figure 7, the element $i_{\mathrm{ab}}$ is loaded into each internal local register via broadcasting for each PE. The k elements of the vector in row B of matrix W are also loaded into each PE to facilitate the vector multiplication and accumulation calculation. The multiplication and accumulation results on each PE are then saved in the on-chip register for use in the next iteration.

Compared with the traditional calculation method, the programming method based on vector SIMD technology is not intuitive. However, conducting the calculation between matrix I and W, row by row, has high memory access efficiency; in particular, it avoids the traditional column vector data access of matrix W, and further does not require transposing the matrix W and calculation sum between PEs. This can reduce the extra cost of inner loop pipelining to the outer loop, and makes it easier to achieve efficient software pipelining.

## 4. Accelerator Architecture Design

### 4.1. Accelerator Architecture Overview

**Figure 8:**
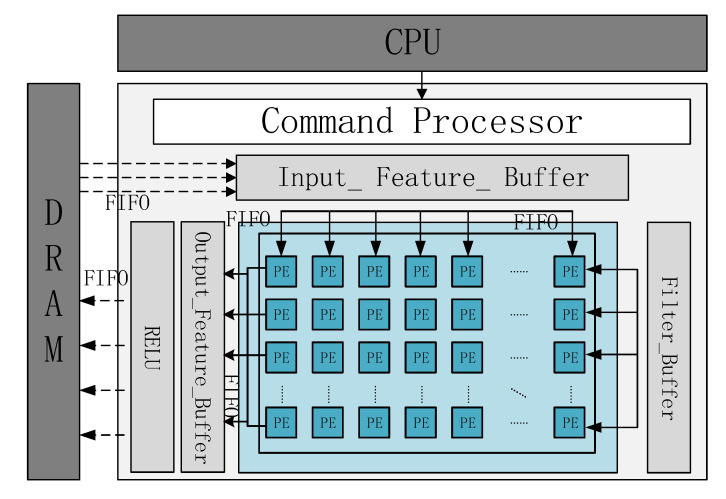
Accelerator structure overview.

The structure of the CNN accelerator is illustrated in Figure 8. It mainly comprises the processor, DRAM (Dynamic Random Access Memory), matrix-multiplication array (MMA), and system interconnection bus.

The main modules and their functions are as follows:

(1) CPU (Central Processing Unit): Runs the program control task execution module, communicates with the accelerator controller through the general interface, and facilitates interaction with the accelerator by writing the task descriptor into the memory mapping register;
(2) Controller: After receiving the task information sent by the CPU, loads the task onto the accelerator and begins the calculation. Distributes the tasks to each MMA execution module according to the present algorithm, and starts the DMA (Direct Memory Access) module through the interconnection configuration to move the data to the BUFFER or write to DDR (Double Data Rate), until the calculation is completed. The results are transferred to the external DRAM, after which the information is sent back to the CPU;
(3) MMA: Calculates the block matrix based on vector SIMD technology.

### 4.2. The Structure of the MMA

**Figure 9:**
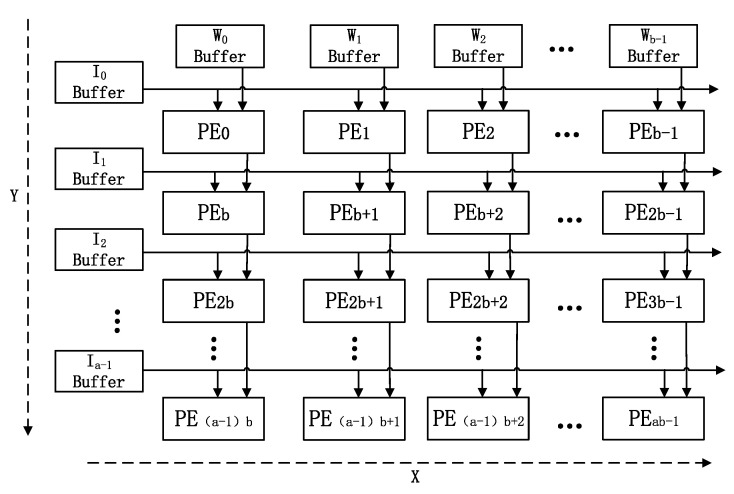
The structure of MMA.

The MMA unit is responsible for the calculation of the block intermediate process matrix. Its overall structure, as shown in Figure 9, consists of a × b processing elements (PEs). This is a structure that combines a systolic array structure and bus broadcast-based parallel structure, and is thus called systolic-based extended parallel architecture. Each PE does not require any data exchange and communication in the x-dimensional direction. Therefore, the bus broadcast mode is used to load data into each PE; this can effectively eliminate data transmission delay between adjacent processing units, and further reduces the difficulty of designing the PE control module in the x-dimensional direction. Moreover, due to data reuse in the Y-dimensional direction, the systolic array structure is adopted to reduce the communication overhead. The PE is connected by short wires, which can effectively ensure the high independence and parallelism of data processing between PEs. This approach can not only reduce the MMA’s bandwidth requirements, but can also solve the problem of the simple bus broadcast structure having insufficient expansibility.

In order to clearly and simply explain the working process of MMA, the process for an example MMA structure with 4 × 4 PE units in processing intermediate process matrix multiplication will be considered: O=IW, the scale of matrix I is 4 × 8, and the scale of matrix W is 8 × 4 (see Equation (4)):

(4) $$\left[\begin{array}{cccc} O_{11} & O_{12} & O_{13} & O_{14}\\ O_{21} & O_{22} & O_{23} & O_{24}\\ O_{31} & O_{32} & O_{33} & O_{34}\\ O_{41} & O_{42} & O_{43} & O_{44} \end{array}\right]=\left[\begin{array}{cccc} I_{11} & I_{12} & \ldots & I_{18}\\ I_{21} & I_{22} & \ldots & I_{28}\\ I_{31} & I_{32} & \ldots & I_{38}\\ I_{41} & I_{42} & \ldots & I_{48} \end{array}\right]\left[\begin{array}{cccc} W_{11} & W_{12} & W_{13} & W_{14}\\ W_{21} & W_{22} & W_{23} & W_{24}\\ \vdots & \vdots & \vdots & \vdots \\ W_{81} & W_{82} & W_{83} & W_{84} \end{array}\right]$$

**Figure 10:**
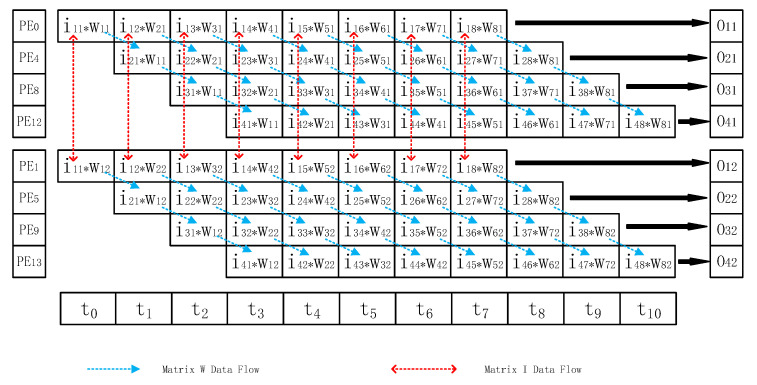
The working flowchart of MMA.

The MMA flow chart is shown in Figure 10. Each PE unit synchronously executes the following process when performing multiplication calculation: first, the data of the filter intermediate matrix W is transmitted to the next PE unit. The adder then adds the last calculation result, with the intermediate result stored on-chip REG in PE and writes it back to the on-chip memory until the end of the cycle. The final result is directly written back to the external memory by the DMA module in the reverse direction of the matrix W input by the systolic array structure.

### 4.3. The Structure of PE

**Figure 11:**
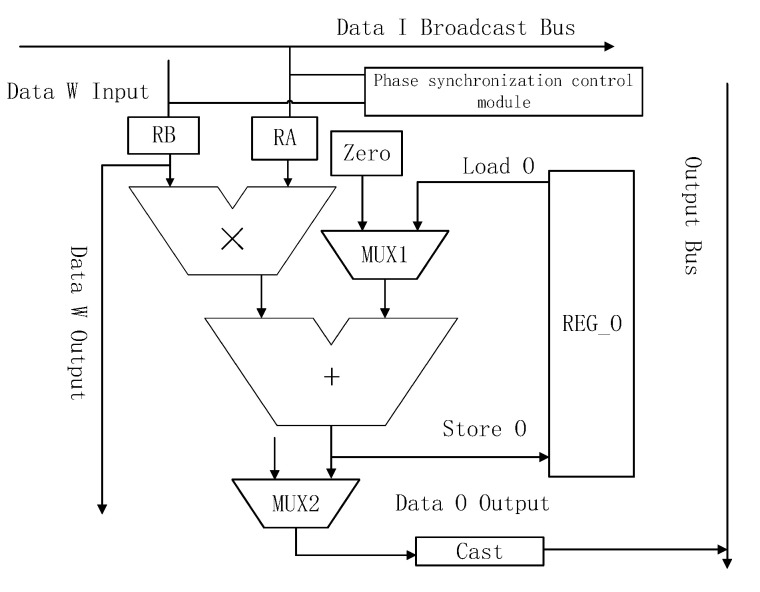
The structure of PE.

A PE is the most basic operation unit of an accelerator. The structure of a PE is presented in Figure 11.

The PE mainly consists of the multiply-add unit, on-chip memory, row and column communication interface, I/O bus, and so on. The PE unit receives the data of matrix I from the broadcast bus and also receives the data of matrix W from the buffer or the previous PE. Only when the data meet the IEEE-754 standard can it be sent to the multiplier. After the multiplication result is obtained, it is input into the adder. The adder reads the intermediate value of the result matrix O from the on-chip register through an MUX1 multiplexer (when the initial calculation is performed, the data of matrix O is zero). After calculation is complete, a MUX2 multiplexer is used to determine whether the result will be stored in on-chip RAM or written to external DRAM. Because the intermediate value is stored in the PE’s internal memory, the calculation is independent, such that there is no need to transfer the intermediate results to an external location.

### 4.4. The Double Buffer Data Movement Based on DMA

**Figure 12:**
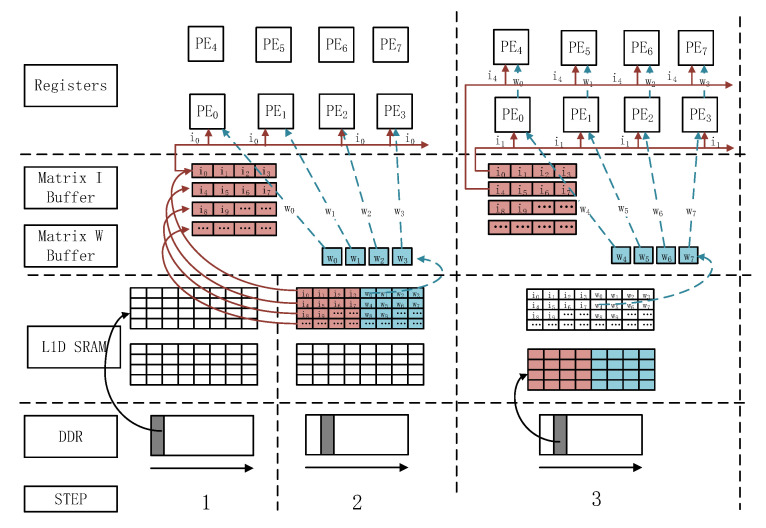
The double buffer data moving strategy based on DMA.

Reducing the cost of moving data between different levels of storage is one of the key means by which the efficiency of matrix-multiplication can be improved. The data storage of an accelerator involves a register file, Buffer, L1D SRAM, and DDR multi-level storage structure. In L1D SRAM, two buffers are set up, while a Ping-Pong relay is used to facilitate the overlapping of core computing and DMA data moving; this is done to reduce the data moving time and improve the calculation efficiency of the CNN accelerator. Taking an MMA with 4 × 4 PEs as an example, the specific data migration strategy is shown in Figure 12.

**Step 1:** Prepare the data of the first buffer in L1D SRAM through DMA before MMA starts the calculation.

**Step 2:** The buffer pools, corresponding to matrix I and matrix W in MMA, read the data in L1D SRAM, respectively. The buffer pools, corresponding to matrix I, read the data of the corresponding rows and transmit them the appropriate PE via bus broadcast. The data of matrix W is read row by row and distributed to each buffer pool corresponding to matrix W, after which the data are transferred to the next PE.

**Step 3:** During the process of data calculation in the PE, DMA moves the next data block of matrix I and matrix W from DDR to the second buffer of L1D SRAM. The matrix W buffer pool reads the remaining data in the first buffer, then uses the MMA’s systolic array structure to transmit the data of matrix W to the next PE via the short line between the PEs. After receiving the data, the PE starts the calculation of the PE data in the second layer. After the PEs have computed the first block matrix operation result, the buffer pools corresponding to matrix I and matrix W continue to read the data of the second buffer in L1D SRAM.

The calculation results of the above process are repeated until each channel of the input feature map is divided into blocks and the corresponding channel data of the filter is calculated; subsequently, the calculation results are moved to DDR by using the systolic structure through DMA.

## 5. Experimental Analysis and Results

### 5.1. Model Analysis

When designing an accelerator, parameters such as memory access ratio and computing performance are important considerations which must be addressed while planning the whole accelerator scale setting, memory setting, and block size of the block algorithm. Suppose the number of arithmetic units in MMA is a×b, the amount of MMA is $n_{\mathrm{MMA}}$, instruction execution time of each PE is $t_{\mathrm{mac}}$ (including calculation and the time required to write the result back to on-chip memory), the matrix W data transmission time between PEs is $t_{W}$, the kernel-level block size of the PE layer is divided into a×k and k×b, and the calculated memory access ratio ƒ can be obtained by Equation (5):

(5) $$f=\frac{flops}{\mathrm{memops}}=\frac{{\mathrm{abk}}^{2}}{\left(a+b\right)k}=\frac{\mathrm{abk}}{a+b}$$

As PE is the smallest block matrix computing unit for computing matrices I and W, the performance of the PE operation will directly affect the calculation of the large-scale matrices I and W. In order to fully guarantee high-efficiency PE calculation, the data transmission bandwidth must meet the calculation needs, as this enables the data transmission time to be hidden in the calculation time. Suppose the data bandwidth between L1D SRAM and DDR is R, while the clock cycle time is *t*; accordingly, the data bandwidth R can be calculated by Equation (6):

(6) $$R>\frac{\left(a+b\right)k\times n_{\mathrm{MMA}}}{a-1t_{W}+t_{\mathrm{mac}}+t\left(k-1\right)}.$$

Since the PE unit can complete one floating-point multiplication and one floating-point addition operation in each clock cycle after the pipeline technology is adopted, the theoretical peak computing performance of the entire CNNs accelerator is thus ${\mathrm{PERF}}_{\max}$, which can be calculated by means of Equation (7):

(7) $${\mathrm{PERF}}_{\max}=\frac{2{\mathrm{ab}*n}_{\mathrm{MMA}}}{t}.$$

The storage cost MI and MK of the intermediate matrix generated by the input feature map and filter respectively can in turn be calculated by Equations (8) and (9), respectively. Finally, the total storage cost MMTCA can be calculated by Equation (10):

(8) $$M_{I}=\left(\frac{W-K+1}{S\times m}+K-1\right)\times K\times \frac{H-K+1}{S}\times m$$

(9) $$M_{K}=\left(\frac{W-K+1}{S\times m}+K-1\right)\times K\times \frac{W-K+1}{S}$$

(10) $$M_{MFCA}=M_{I}+M_{K}.$$

By deriving Equation (10) on M, Equation (11) can be obtained, as follows:

(11) $${\;M\prime }_{MFCA}=\left(K-1\right)\times K\times \frac{H-K+1}{S}-\frac{K{\left(W-K+1\right)}^{2}}{S^{2}\times m^{2}}$$

The best block number with minimum storage consumption based on the MTCA algorithm for different convolution types can therefore be expressed by Equation (12):

(12) $$m=\lceil \frac{W-K+1}{S}\sqrt{\frac{S}{\left(K-1\right)\left(H-K+1\right)}}\rceil$$

### 5.2. Experimental Results and Discussion

**Figure 13:**
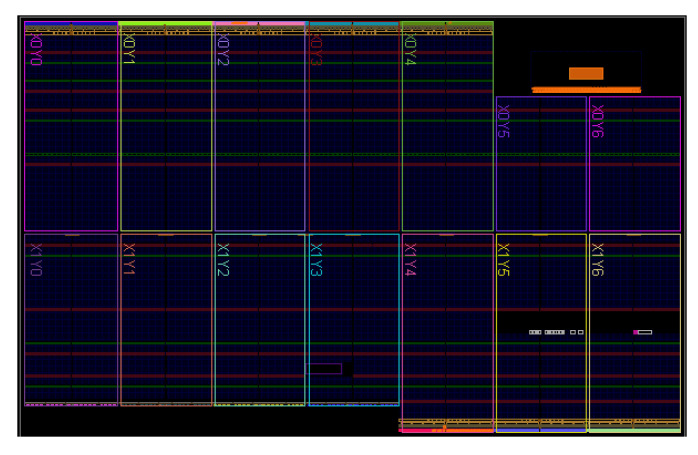
Gate-level Netlist generated in Vavido.

Figure 13 shows the synthesized gate level network table generated in Vivado. This design uses Modelsim 10.2 to test the function and timing of the complete design. The generic cabling tool uses the Xilinx Vivado 2016.2 toolchain to realize an (8 × 8) × 2 scale accelerator design on FPGA. The peak performance reaches 38.4GFLOPS, on-chip power is 2.95 W, and per power consumption performance reaches 13GFLOPS.

In order to facilitate the desired analysis, we use the different convolution types and the best MTCA block number provided in Table 1 for our experiments.

**Figure 14:**
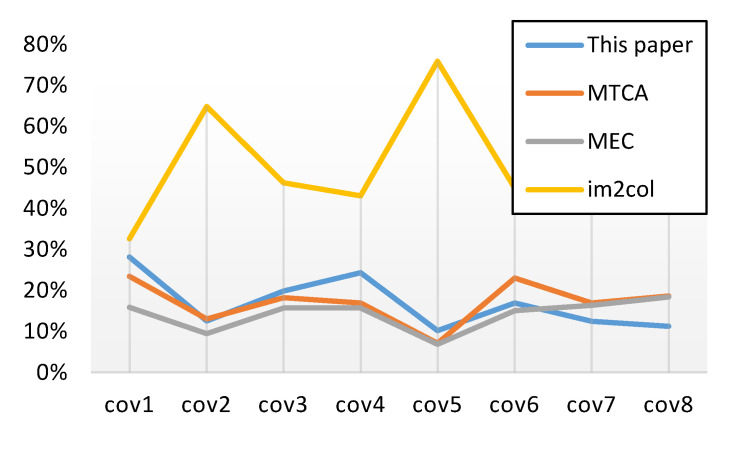
The storage overhead comparison between different convolution algorithms.

Figure 14 describes the consumption of storage resources when the MTCA block parallel computing algorithm, MTCA, MEC, and *im2col* are used to test the testing set. From the figure, it can be seen that the MTCA block parallel computing algorithm and the MTCA do not dominate in terms of performance when the size of the filter or input feature map is small. In the case of small convolution size, MEC consumes the lowest amount of hardware storage resources and saves about 50% storage space compared with the traditional *im2col* algorithm. However, the block logic of MEC is complex; this is because the address space of the intermediate process matrix is not aligned, making it very complex and difficult to implement on hardware. For its part, the MTCA block parallel computing algorithm saves more than 60% of storage space compared with the traditional algorithm. Furthermore, when the convolution size is large, the MTCA block parallel computing algorithm exhibits more obvious advantages than others (e.g., cov7 and cov8). This proves that the parallel computing algorithm is not only conducive to parallel computing, but can also save space when calculating large-scale convolutions.

**Figure 15:**
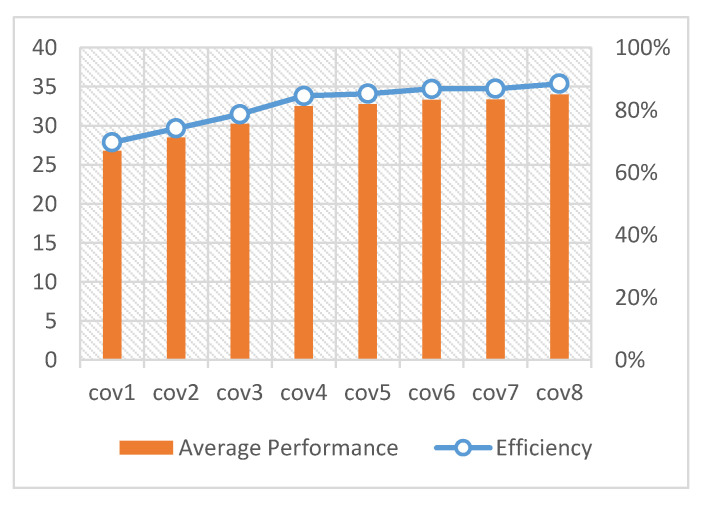
Accelerator computing performance and efficiency.

Figure 15 illustrates the computational efficiency of the CNN accelerator under different convolution types. It can be seen from the figure that when the size of the matrix is small, the calculation efficiency is low; this is because even if the optimization strategy of DMA-based double buffer data migration is adopted in order to hide the data handling time in the data calculation, the DMA channel start time, the time of the first data handling, and the time of the last data writing cannot be hidden in this way. At the same time, moreover, the time of the first convolution expansion and the writing time cannot be hidden, which reduces the computational efficiency of the entire accelerator. When the convolution size is small, the time required for convolution expansion and for intermediate matrix reading and writing will be longer than the corresponding calculation time; this means that the data handling time can no longer be hidden between the operations, resulting in overall performance degradation.

**Figure 16:**
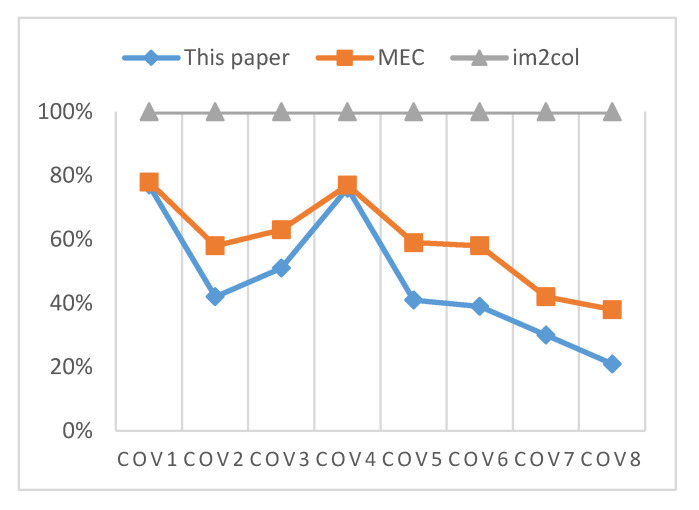
The running time between different convolution conversion algorithms.

Figure 16 describes the convolution operation time under different convolution conversion algorithms, which are compared with the running time required by im2col. The figure shows that the MTCA block parallel computing algorithm achieves minimal performance improvement compared with EMC in the case of small convolution size. As the convolution size increases, the MTCA block parallel computing algorithm has certain advantages. Compared with EMC, the MTCA block parallel computing algorithm can improve the acceleration effect by about 20% and can achieve more than three times of im2col.

**Figure 17:**
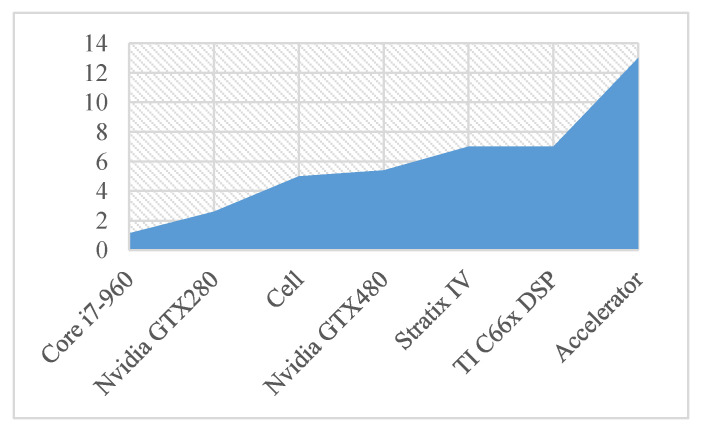
Per unit power operation Performance of different processors handling cov8.

It can be seen from Figure 17 that the per power performance of Core i7-960 is the worst, followed by NVIDIA series, while the accelerator we designed has advantages in per unit power operation performance, and the per unit power operation performance reaches 13 GFLOPS/W.

## 6. Conclusions

In this paper, we propose a design based on the MTCA block parallel computing algorithm for CNNs hardware accelerator. With the row matrix-multiplication computing, the MTCA block parallel computing algorithm can provide high performance and high efficiency acceleration for various convolutions on the premise of reducing bandwidth and maximizing data reuse. In our design, we realize the convolution expansion and the partition of the intermediate matrix, which is conducive to the high parallel computation on hardware. Compared with the accelerator design based on the *im2col* algorithm, our design can save more than 60% of the hardware storage space. Especially in the case of computing large-scale convolution, it saves nearly 82% of the storage space. In addition, it shows more prominent advantages than the accelerator design based on MEC. Experiments show that, with the support of specific dataflow mode, and the per unit power operation performance reaches 13 GFLOPS/W, which is better than popular processors.

## Figures and Tables

**Table 1:** Various types of convolutions and their optimal number of blocks.

		Input Feature Map	Filter	Optimal m
COV1	ResNet-18 COV5_X	7 × 7	3*3	2
COV2	ResNet-18 COV4_X	24 × 24	3*3	6
COV3	ResNet-18 COV2_X	56 × 56	3*3	7
COV4	ResNet-18 COV1_X	112 × 112	3*3	3
COV5	VGG-16 COV1	224 × 224	3*3	5
COV6	AlexNet COV1	227 × 227	11*11	16
COV7		720 × 480	5*5	9
COV8		1920 × 1080	5*5	12

Each network parameter comes from the official open literature.

## References

[1] Krizhevsky A., Sutskever I., Hinton G.E. (2012). ImageNet classification with deep convolutional neural networks. Adv. Neural Inf. Process. Syst..

[2] Dong G., Liu H., Kuang G., Chanussot J. (2019). Target recognition in SAR images via sparse representation in the frequency domain. Pattern Recognit..

[3] Uijlings J.R., VandeSande K.E.A., Gevers T., Smeul-ders A.W.M. (2013). Selective search for object recognition. Int. J. Comput. Vis..

[4] Girshick R., Donahue J., Darrell T., Malik J. (2014). Rich feature hierarchies for accurate object detection and semantic segmentation. Proc. IEEE CVPR.

[5] Noh H., Hong S., Han B. (2015). Learning deconvolution net-work for semantic segmentation. Proc. IEEE ICCV.

[6] Liu S., Du Z., Tao J. (2016). Cambricon: An instruction set architecture for neural networks. ACM Sigarch Comput. Archit. News.

[7] Lavin A., Gray S. (2016). Fast algorithms for convolutional neural net-works. Proc. IEEE CVPR.

[8] Chen Y.H., Krishna T., Emer J.S., Sze V. Eyeriss: A Spatial Architecture for Energy-Efficient Dataflow for Convolutional Neural Networks. Proceedings of the 2016 ACM/IEEE 43rd Annual International Symposium on Computer Architecture (ISCA).

[9] Yin S. (2018). A high energy efficient reconfigurable hybrid neural network processor for deep learning applications. IEEE J. Solid-State Circuits.

[10] Desoli G. A 2.9TOPS/W deep convolutional neural network SoC in FD-SOI 28 nm for intelligent embedded systems. Proceedings of the IEEE Int. Solid-State Circuits Conference (ISSCC).

[11] Shin D., Lee J., Yoo H.J. DNPU: An 8.1TOPS/W reconfigurable CNN-RNN processor for general-purpose deep neural networks. Proceedings of the IEEE International Solid-State Circuits Conference (ISSCC).

[12] Wang J., Lin J., Wang Z. (2018). Efficient hardware architectures for deep convolutional neural network. IEEE Trans. Circuits Syst. I.

[13] Ma Y., Cao Y., Vrudhula S., Seo J.S. (2018). Optimizing the convolution operation to accelerate deep neural networks on FPGA. IEEE Trans. Very Large Scale Integr. (VLSI) Syst..

[14] Ardakani A., Condo C., Ahmadi M., Gross W.J. (2018). An architecture to accelerate convolution in deep neural networks. IEEE Trans. Very Large Scale Integr. (VLSI) Syst..

[15] Fang Y., Chen Q. (2019). Optimization method of convolution calculation based on matrix transformation. Comput. Eng..

[16] Kung H.T., Leiserson C.E. (1978). Systolic Arrays. Handbook of Signal Processing Systems.

[17] Chen T., Du Z., Sun N. (2014). DianNao: A small-footprint high-throuhput accelerator for ubiquitous machine-learning. ACM SIGARCH Comput. Archit. News.

[18] Chen Y., Lou T., Liu S. (2014). DaDianNao: A machine-learning supercomputer. ACM Int. Symp. Microarchit..

[19] Chen Y.H., Krishna T., Emer J.S., Sze V. (2017). Eyeriss: An energy-efficient reconfigurable accelerator for deep convolutional neural net-works. IEEE J. Solid-State Circuits.

[20] You H., Junzhon S., Yuran Q. (2018). MALMM: A Multi-array Architecture for Large-scale Matrix Multiplication on FPGA. IEICE Electron. Express.

[21] Zhang J.Y., Guo Y., Hu X. (2018). Parallel computing method of two-dimensional matrix convolution. Eng. Sci..

[22] Jing S., Haoqi R., Zhifeng Z., Jun W., Zhenyu J. A High-Performance Systolic Array Accelerator Dedicated for CNN. Proceedings of the 2019 IEEE 19th International Conference on Communication Technology (ICCT).

[23] Chaoyang Z., Kejie H., Shuyuan Y., Ziqi Z., Hejia Z. (2020). An Efficient Hardware Accelerator for Structured Sparse Convolutional Neural Networks on FPGAs. IEEE Trans. Very Large Scale Integr. (VLSI) Syst..

[24] Maurizio C., Beatrice B., Alberto M., Muhammad S. (2020). An Updated Survey of Efficient Hardware Architectures for Accelerating Deep Convolutional Neural Networks. Future Internet.

[25] Cho M., Brand D. MEC: Memory-efficient convolution for deep neural network. Proceedings of the 34th International Conference on Machine Learning.

[26] Liu Z., Tian X. (2018). Matrix multiplication and vectorization for multi-core vector processors. J. Comput. Sci..

